# The Lysosome Signaling Platform: Adapting With the Times

**DOI:** 10.3389/fcell.2019.00113

**Published:** 2019-06-20

**Authors:** Subothan Inpanathan, Roberto J. Botelho

**Affiliations:** Department of Chemistry and Biology, Graduate Program in Molecular Science, Ryerson University, Toronto, ON, Canada

**Keywords:** organelles, compartmentalisation, intracellular signaling, cellular adaptation, stress, inflammation, infection, metabolism

## Abstract

Lysosomes are the terminal degradative compartment of autophagy, endocytosis and phagocytosis. What once was viewed as a simple acidic organelle in charge of macromolecular digestion has emerged as a dynamic organelle capable of integrating cellular signals and producing signal outputs. In this review, we focus on the concept that the lysosome surface serves as a platform to assemble major signaling hubs like mTORC1, AMPK, GSK3 and the inflammasome. These molecular assemblies integrate and facilitate cross-talk between signals such as amino acid and energy levels, membrane damage and infection, and ultimately enable responses such as autophagy, cell growth, membrane repair and microbe clearance. In particular, we review how molecular machinery like the vacuolar-ATPase proton pump, sestrins, the GATOR complexes, and the Ragulator, modulate mTORC1, AMPK, GSK3 and inflammation. We then elaborate how these signals control autophagy initiation and resolution, TFEB-mediated lysosome adaptation, lysosome remodeling, antigen presentation, inflammation, membrane damage repair and clearance. Overall, by being at the cross-roads for several membrane pathways, lysosomes have emerged as the ideal surveillance compartment to sense, integrate and elicit cellular behavior and adaptation in response to changing environmental and cellular conditions.

## Introduction

**FIGURE 1:**
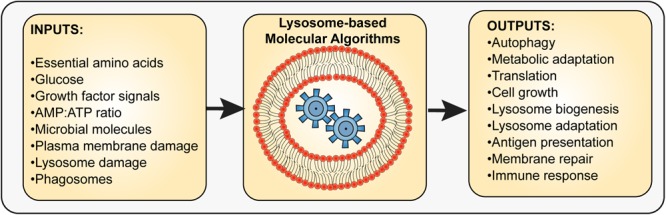
Input and outputs integration by the lysosome. The lysosome interfaces with multiple molecular sensors that sense the levels of specific metabolites such as amino acids, glucose, and AMP, extracellular cues such as growth factors, hormones, and microbe-derived molecules, and stress indicators such as those released by membrane damage. The integration of sensors and the molecular pathways used to process these inputs then lead to output responses that aid in cell survival, adaptation, or stress resolution. These outputs may include autophagy regulation, metabolic adaptation, altered protein synthesis and turnover rates, antigen processing and presentation, and lysosome exocytosis, among other possible responses. Thus, the lysosome is a key integrator and organizer of cellular adaptation and survival.

Since their discovery by Christian de Duve, lysosomes have classically been thought to provide a “janitorial” service to cells by digesting unwanted macromolecules, damaged organelles, microbes and other particulates delivered via endocytosis, autophagy, and phagocytosis (de Duve et al., 1955; Settembre et al., 2013; Luzio et al., 2014; Hipolito et al., 2018). As with real-world janitorial services, lysosomes were appreciated for their essential role in maintaining cellular homeostasis, but this acidic and degradative terminal organelle arguably lacked “glamor.” However, over the past 60 years, our view of the lysosome has evolved from a simple, degradative organelle to a dynamic hub capable of detecting and interpreting cellular signals to produce and execute downstream responses. The list of functions now assigned to lysosomes include membrane repair, nutrient and energy homeostasis, modulating gene expression, maintaining neurophysiology, processing and presenting antigens, and regulating a variety of other immune responses, among many others (Lim and Zoncu, 2016; Hesketh et al., 2018; Hipolito et al., 2018; Lamming and Bar-Peled, 2018; Lie and Nixon, 2018). Perhaps central to this evolving picture is the connection between lysosomes and the ever important mTORC1, which uses the lysosome as a signaling platform, integrating nutrient and energy cues to govern the balance between catabolic and anabolic processes within the cell (Lim and Zoncu, 2016). Beyond this, lysosomes serve as a scaffold for AMPK, which counteracts mTORC1, and glycogen synthase kinase-3β (GSK3β), which further integrates growth and apoptotic signals, and even transcription factors such as TFEB that promotes the expression of lysosomal genes in an effort to boost lysosome function in response to stresses like starvation and infection (Sardiello, 2016; Carroll and Dunlop, 2017; Bautista et al., 2018). In this review, we discuss past and recent advancements that place lysosomes as major organizers of cellular signaling, mapping out key pathways that the lysosome integrates to govern diverse cellular functions. We begin with a short synopsis written at a high-level to position the lysosome within the endomembrane system, citing mostly recent reviews on specific subjects to guide the reader to greater detail on these introductory topics. We then highlight specific molecular sensor circuits that lysosomes are equipped with, followed by an examination of the responses elicited by this molecular circuitry (Figure 1). We note that we use the term *lysosome* to refer to a spectrum of organelles that include late endosomes, terminal lysosomes, and endolysosomes, the latter describing late endosome-lysosome hybrids (Bright et al., 2016).

## The Lysosome Is at the Cross-Roads of Major Trafficking Routes

### A Beginner’s Guide to the Endo-Lysosomal Membrane System

**FIGURE 2:**
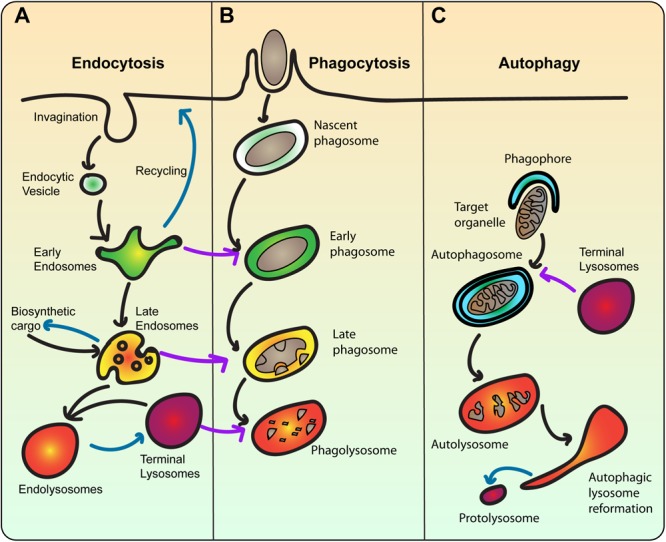
The lysosome is the terminal compartment for endocytosis, phagocytosis, and autophagy. **(A)** During endocytosis, plasma membrane invaginates to form endocytic vesicles that contain extracellular fluid and membrane cargo. Endocytic vesicles then fuse with early endosomes, which sort cargo for recycling back to the plasma membrane or degradation towards lysosomes. Concurrent with sorting, early endosomes mature into multivesicular bodies that then become late endosomes. Late endosomes also receive newly synthesized cargo including lysosomal proteases. Late endosomes then fuse with terminal lysosomes, which are non-acidic stores of hydrolytic enzymes to form a hybrid endolysosome, where degradation ensues. Endolysosomes may be able to reform terminal lysosomes. Blue arrows indicate recycling/reformation pathways. **(B)** In phagocytosis, extracellular particles like bacteria are engulfed by the plasma membrane and sequestered within a phagosome. Phagosomes are then thought to mature by sequentially fusing with early and late endosomes, and ultimately lysosomes. This transforms the nascent phagosome from an innocuous organelle into an acidic and degradative phagolysosome, where the particle is digested. The ultimate fate of the phagolysosome is enigmatic. As such, the endo-lysosomal pathway is a template for phagosome maturation. **(C)** In autophagy, cytoplasmic material like damaged or surplus organelles is targeted for entrapment by the phagophore, a double-bilayer membrane derived from the ER, forming the autophagosome. Akin to phagosomes, autophagosomes also experience a maturation process, ultimately fusing with lysosomes. Upon degradation of cargo, autolysosomes undergo autophagic lysosome reformation, whereby tubular membrane extrusions extrude proto-lysosomes that reform lysosomes consumed during autophagy.

Lysosomes are the common terminal nexus of endocytosis, phagocytosis, autophagy and biosynthetic routes, receiving both extracellular and intracellular-derived molecular cargo, cytoplasmic cargo like damaged organelles, and engulfed dead cells and foreign particulates like bacteria for digestion (Figure 2). During endocytosis, extracellular and plasma membrane molecules are internalized via several mechanisms such as clathrin-mediated endocytosis that enrich cargo within plasma membrane subdomains that then invaginate and ultimately undergo scission to release an endocytic vesicle (Kirchhausen et al., 2014). Fluid-phase cargo is non-specifically trapped within the emerging bud, while cargo that binds cognate receptors is enriched within the nascent vesicles. Cargo can include nutrient delivery systems like LDLs that delivers dietary cholesterol, and transferrin that delivers protein-bound iron. Additionally, cargo can comprise a variety of signaling receptors bound to hormones, growth factors, or mitogens, serving to down-regulate signaling after an initial burst by abating receptor levels on the plasma membrane (Kirchhausen et al., 2014). Lastly, endocytosis helps to remodel the plasma membrane proteome by removing transporter proteins and cell adhesion molecules in response to specific triggers (Ross et al., 2015). Regardless of mechanism and cargo, endocytic vesicles contain molecular information in the form of specific Rab GTPases and SNAREs to then fuse with early endosomes.

Early endosomes are a collection of endosomal compartments whose job is to sort the endocytosed cargo for either direct recycling to the plasma membrane, or via recycling endosomes, traffic to the *trans*-Golgi network, or to late endosomes for ultimate degradation in lysosomes (Naslavsky and Caplan, 2018). The canonical early endosome is identified by the Rab5 GTPase, which facilitates tethering and fusion to endocytic vesicles and with other early endosomes. Rab5 recruits Vps34, a phosphatidylinositol 3-kinase to produce the phosphatidylinositol-3-phosphate [PtdIns(3)P] lipid. Rab5 and PtdIns(3)P then recruit several effectors such as the early endosomal antigen 1 (EEA1), which tethers endosomes together to mediate fusion via specific SNAREs (Simonsen et al., 1998, 1999; Christoforidis et al., 1999; Dumas et al., 2001; Naslavsky and Caplan, 2018). A variety of complexes comprising coat proteins, PtdIns(3)P-binding sorting nexins, and acto-myosin machinery drive recycling of specific cargo like transferrin receptors by forming specialized endosomal tubules that eventually bud to transport cargo to the plasma membrane, the Golgi, or recycling endosomes (Carlton et al., 2004; Derivery et al., 2009; Coudrier and Almeida, 2011; Gautreau et al., 2014; Naslavsky and Caplan, 2018). While recycling cargo is extruded through vesiculation and tubulation, cargo destined for degradation like ubiquitylated signaling receptors are entrapped within endosomal subdomains by the ESCRT proteins that inversely deform the membrane domain towards the lumen of the endosome to generate an intraluminal vesicle (Migliano and Teis, 2018; Szymanska et al., 2018). This process effectively matures early endosomes into multivesicular bodies that then fuse or become late endosomes, themselves enriched in intraluminal vesicles. Overall, this transition is characterized by replacing Rab5 with the Rab7 GTPase, a hallmark process in early to late maturation of endosomes (Rink et al., 2005; Huotari and Helenius, 2011).

When active, the Rab7 GTPase regulates the position of late endosomes by interfacing with microtubule-dependent dynein via RILP and kinesins via FYCO1 (Cantalupo et al., 2001; Jordens et al., 2001; Pankiv et al., 2010; Pu et al., 2016). This is coordinated with tethering and fusion functions modulated by complexes like the homotypic fusion and protein sorting (HOPS) and SNAREs (Balderhaar and Ungermann, 2013; Kümmel and Ungermann, 2014). Late endosomes also receive newly synthesized lysosomal membrane proteins and hydrolases that originate from the ER and the Golgi network (Naslavsky and Caplan, 2018). Thus, late endosomes are a node that intermixes endocytic and biosynthetic cargo. They are also equipped with fission complexes like retromer to recycle receptors that transport biosynthetic cargo back to the Golgi (Naslavsky and Caplan, 2018). Ultimately, late endosomes are thought to fuse with lysosomes.

Lysosomes are enriched in >50 hydrolytic enzymes and display a luminal pH < 5, established by the vacuolar-ATPase (V-ATPase) H^+^ pump, thus gathering the reputation of being the cell’s hydrolytic center (Mindell, 2012; Luzio et al., 2014). Lysosomes act as a store for additional ions and metabolites including Ca^2+^, phosphate, ATP, and Zn^2+^ (Blaby-Haas and Merchant, 2014; Lim and Zoncu, 2016). Depending on the cell type, lysosomes can range in the dozens to hundreds to form a heterogeneous population in terms of degradative activity, luminal pH, cargo access, shape and subcellular distribution. For example, perinuclear lysosomes tend to be more acidic, while peripheral lysosomes are more alkaline (Johnson et al., 2016). Lysosome position within the cell is established in coordination with Rab7 and Arl8b GTPases; as noted above, Rab7-RILP regulates lysosomal movement by recruiting dynein-dynactin motor complexes and facilitates movement towards the cell center by following the minus-ends of microtubules (Cantalupo et al., 2001; Jordens et al., 2001). In comparison, Arl8b uses the SKIP effector to bind kinesin to promote movement towards the cell periphery by following the plus-end of microtubules (Rosa-Ferreira and Munro, 2011; Pu et al., 2015). Interestingly, these activities are also able to shape lysosomes, which are typically spherical organelles, into tubular networks in activated immune macrophages and dendritic cells (Mrakovic et al., 2012; Saric et al., 2016; Hipolito et al., 2018).

Strikingly, it is challenging to differentiate late endosomes from lysosomes based on commonly employed markers like Rab7 and LAMP1. Partly, this is because these organelles form a continuum rather than a defined organelle group. Indeed, a more refined view of the late endosome-lysosome system categorizes these organelles into at least three classes (Bright et al., 2016). Late endosomes as defined above carry cargo for degradation and newly synthesized cargo. In contrast, terminal lysosomes are enriched in lysosomal proteins and hydrolases but are not hydrolytically active, acting as a storage organelle. However, late endosomes can then fuse with terminal lysosomes either completely or transiently through kiss-and-run interactions to generate endolysosomes, a late endosome-lysosome hybrid where degradation ensues (Bright et al., 2016; Bissig et al., 2017; Figure 2). Terminal lysosomes themselves can be regenerated during kiss-and-run or through budding events (Bright et al., 2016; Bissig et al., 2017). Relative to trafficking mechanisms to lysosomes, lysosome vesiculation and recycling is much less understood, though it involves classical coat proteins like clathrin and dynamin and putative vesiculation proteins like spastizin, Atg18, and actin-based machinery (Saffi and Botelho, 2019).

### Lysosomes Interface With Phagocytosis and Autophagy

The description above charts the canonical endo-lysosomal system, which then serves as a template for phagosome and autophagosome maturation. Phagocytosis relies on phagocytic receptors that bind ligands decorating a target particle such as apoptotic bodies or microbes to elicit the engulfment and sequestration of the particle within a phagosome (Levin et al., 2016; Gray and Botelho, 2017; Niedergang and Grinstein, 2018). To illustrate, bacteria and fungi, respectively, display conserved microbe-associated molecular patterns like LPS and β-glucans, which are then, respectively, recognized by phagocytic receptors CD14 and dectin-1 (Ishiguro et al., 2001; Schorey and Lawrence, 2008); alternatively, microbes can be decorated by cognate antibodies like immunoglobin G (IgG) that then engage Fcγ receptors (Levin et al., 2016; Gray and Botelho, 2017; Niedergang and Grinstein, 2018). Regardless of receptor-ligand pair, engagement of phagocytic receptors leads to signal transduction pathways that remodel the underlying plasma membrane via actin polymerization and localized exocytosis, forming a phagocytic cup around the bound particle (Gordon, 2016; Levin et al., 2016; Gray and Botelho, 2017).

The nascent phagosome then pinches and is released into the cytoplasm sequestering the particle – the incipient phagosome is innocuous but is programmed to fuse with early endosomes, transiently acquiring their properties (Figure 2). These are then divested and replaced by properties that mimic late endosomes and ultimately lysosomes, culminating in the phagolysosome – a highly hydrolytic and acidic organelle that degrades the enclosed particle (Levin et al., 2016; Gray and Botelho, 2017). Phagosomes are also capable of recycling and extruding content, a key process for antigen presentation (Mantegazza et al., 2013, 2014). However, there is very little known about the ultimate fate of phagolysosomes, post-particle degradation (Levin et al., 2016). The typical view is that phagolysosome remnants are secreted, which is based on free-swimming amoeba and protists (Guerrier et al., 2017). However, we venture that this is not likely to occur in multicellular organisms and instead phagosomes are likely resorbed into the endomembrane system; this is evinced by work showing that mTORC1 and the phosphatidylinositol-3-phosphate 5-kinase PIKfyve are required to shrink and fragment lysosomes (Krajcovic et al., 2013; Krishna et al., 2016).

Finally, autophagy delivers cytoplasmic cargo to lysosomes rather than extracellular cargo. Generally, surplus organelles, damaged organelles, protein aggregates, and free bacteria that escape phagosomes are targeted for autophagy (Casanova, 2017; Condello et al., 2019; Zhao and Zhang, 2019). For example, amino acid starvation forces many energy and biosynthesis pathways to idle; consequently, mitochondria and ribosomes are targeted for digestion to regenerate raw materials via mitophagy and ribophagy (Condello et al., 2019).

Autophagy is generally controlled by mTORC1 and AMPK activities in converse. For example, in nutrient replete conditions, mTORC1 is active and AMPK inactive, which represses autophagy; conversely, nutrient depletion inactivates mTORC1 and stimulates AMPK, which promotes autophagy – we will cover in more detail this regulatory network below (Lim and Zoncu, 2016; Carroll and Dunlop, 2017; Condello et al., 2019). Cargo destined for autophagy is demarcated by ubiquitylation or by other adaptor proteins like LC3-II that are then recognized by autophagic receptors like the p62 sequestosome (Condello et al., 2019). These receptors then target cargo to the ER to form the omegasome, a double-bilayer structure derived from the ER that grows and seals the cargo within a double-bilayered autophagosome (Condello et al., 2019; Zhao and Zhang, 2019). Autophagosomes then mature via multiple fusion events with varying stages of the endo-lysosomal system via Rab GTPases, tethering factors and SNARE complexes to transform the autophagosome into an autolysosome (Yu and Melia, 2017; Zhao and Zhang, 2019). For example, Rab7 via its effectors PLEKHM1, FYCO1 and the HOPS complex tether lysosomes to autophagosomes by binding LC3, an autophagosomal membrane protein, which ultimately leads to lysosome-autophagosome fusion (Pankiv et al., 2010; McEwan et al., 2015). After digestion, the spent autolysosomes undergo a fragmentation phenomenon called ALR, whereby lysosomes are regenerated (Yu et al., 2010; Chen and Yu, 2017). We will revisit some of these topics in greater detail below. We next elaborate on the key aim of our review, which is to discuss how lysosomes serve to integrate sensors and signaling outcomes in response to environmental cues and stresses.

## The Lysosome as a Sensor for Cellular Stresses

### The Lysosome Is a Platform for Signaling Complexes

As the primary site of cellular digestion, lysosomes support cell function by recycling and supplying a pool of valuable building blocks like amino acids, saccharides, lipids, ions and nucleobases. In addition, as the terminal organelle of endocytosis, phagocytosis and autophagy, the lysosome is well-positioned to surveil and survey the state of the cell, including incoming nutrient and energy levels and the presence of dangerous factors like microbe-associated molecular patterns. Thus, lysosomes can inform the rest of the cell of its intracellular and extracellular milieu and trigger pathways to help the cell adapt to emerging conditions. This is possible because lysosomes serve as platforms to assemble signaling hubs like mTORC1, AMPK, and GSK3β on their cytosolic surface (Zhang et al., 2013; Carroll and Dunlop, 2017; Bautista et al., 2018; Hesketh et al., 2018; Lamming and Bar-Peled, 2018). We start with a brief illustration of this concept by focusing on the mTORC1 complex and assembly on lysosomes.

**FIGURE 3:**
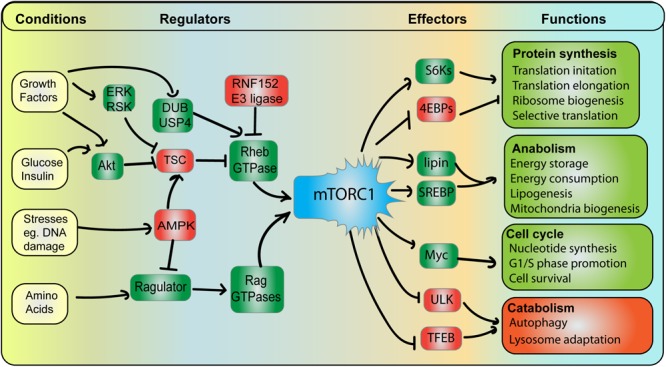
mTORC1 integrates and coordinates multiple signals and responses. *Conditions*: mTORC1 activity is subjected to various cellular conditions, whereby pro-growth triggers (e.g., growth factors, glucose, and amino acids) promote mTORC1, while stress conditions (e.g., DNA damage) abate mTORC1 activity. *Regulators*: Growth conditions stimulate mTORC1 by (i) arresting the Rheb GAP, TSC, leading to GTP-bound Rheb GAP and (ii) stimulating Rag GTPase GEF complex, Ragulator, leading to GTP-bound RagA/B. See text for additional detail. Among negative regulators, AMPK acts as a major dampener of mTORC1 function by concurrently abating Ragulator and promoting TSC, thus hindering Rag and Rheb GTPases. *Effectors and Functions*: Active mTORC1 can then phosphorylate a variety of effector proteins. This includes activating S6Ks but blocking 4E-BP proteins, which ultimately promotes protein synthesis by increasing the rate of mRNA translation initiation and elongation, ribosome biogenesis and selective translation of specific mRNAs. mTORC1 also promotes lipid biosynthesis, energy storage and consumption by stimulating lipin-1 and SREBP, which enhance expression and activity of lipid biogenesis enzymes. mTORC1 stimulates Myc among other targets to drive cells towards growth and mitosis. In comparison, mTORC1 blocks ULK1 and TFEB to arrest catabolic and degradative pathways including autophagy and lysosome biogenesis to elicit biomass accumulation.

Mechanistic target of rapamycin complex 1 is a protein complex composed of five key subunits: four structural and regulatory subunits (Raptor, PRAS40, Deptor, and mLst8) and the conserved mTOR Ser/Thr kinase (Zheng et al., 2014; Rabanal-Ruiz and Korolchuk, 2018). This is in comparison to mTORC2, which contains Rictor and mSin1 rather than Raptor and PRAS40. Interestingly, mTORC2 is an upstream stimulant of Akt, which as described below, promotes mTORC1 activity (Linke et al., 2017; Kim and Guan, 2019). The role of mTORC1 is to foster anabolic and growth functions by stimulating proteins, lipid and nucleotide synthesis, all while suppressing catabolic functions like autophagy and degradation (Laplante and Sabatini, 2012; Lim and Zoncu, 2016; Kim and Guan, 2019; Figure 3). mTORC1 accomplishes these functions by phosphorylating numerous effectors. For instance, mTORC1 promotes ribosome biogenesis by phosphorylating and promoting S6 kinases and enhancing translation of ribosome encoding mRNAs (Brown et al., 1995; Iadevaia et al., 2014); in addition, mTORC1 boosts translation initiation of 5′-cap mRNAs by phosphorylating and arresting the inhibitory translation initiation factors, the 4E-binding proteins (4E-BPs) (Hara et al., 1997; Lim and Zoncu, 2016). Secondly, mTORC1 promotes lipid synthesis by stimulating lipin-1 and the SREBP1/2 transcription factors to augment function and expression of lipid metabolizing enzymes (Porstmann et al., 2008; Peterson et al., 2011). Lastly, mTORC1 promotes nucleotide synthesis to prepare cells for cell division by stimulating the Myc transcription factor (Valvezan et al., 2017). Meanwhile, mTORC1 phosphorylates and represses ULK1 and the TFEB transcription factor to, respectively, abate autophagy initiation and lysosome degradative power (Hosokawa et al., 2009; Kim et al., 2011; Roczniak-Ferguson et al., 2012; Settembre et al., 2012; Martina and Puertollano, 2013). Thus, mTORC1 governs critical functions that underpin cell growth and health – with the lysosome being the platform that organizes its regulation and function.

**FIGURE 4:**
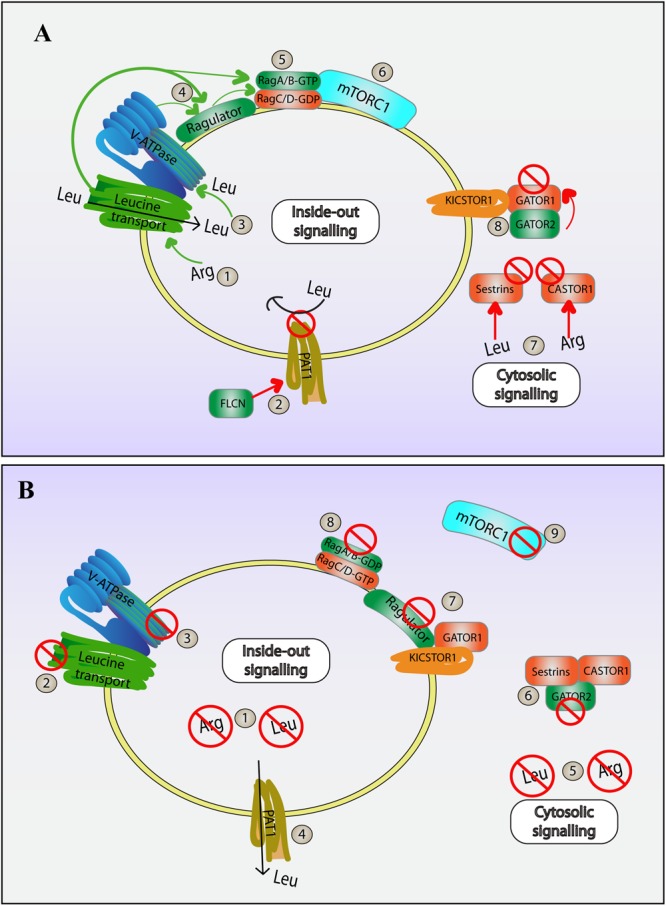
Amino acid dependent regulation of mTORC1. mTORC1 activity is modulated by the levels of specific amino acids within the lumen of lysosomes and in the cytosol. Information about luminal amino acids is transduced by an inside-out mechanism to mTORC1 present on the cytosolic surface, while cytosolic amino acid levels is sensed by cytosolic complexes that associate with the lysosome surface to modulate mTORC1. The following is a simplified series of steps that modulate mTORC1 both during high **(A)** and low **(B)** levels of leucine and arginine. **(A)** High amino acid conditions: (1) High levels of intraluminal arginine promotes leucine transport into the lysosome via SLC38A9. (2) FLCN-mediated inhibition of PAT1 amino acid transporter staunches leucine flow out of the lysosome, helping to amass leucine within the lysosome further; (3) Higher levels of leucine promote V-ATPase activity. (4) V-ATPase and SLC38A9 then promote Ragulator and interactions with Rag heterodimers. (5) Ragulator is a GEF for RagA/B, and together with folliculin, a GAP for RagC/D, helps form GTP-RagA/B::GDP-RagC/D heterodimer. (6) Active Ragulator and Rag heterodimers now recruit and activate mTORC1 on the lysosome surface. (7) Meanwhile, in the cytosol, cytosolic leucine and arginine bind to and inhibits sestrins and CASTOR1, respectively. This prevents sestrins and CASTOR from compromising GATOR2. (8) Ultimately, this allows for GATOR2 to bind and block GATOR1, a negative modulator of mTORC1. Together, both cytosolic and luminal amino acid sensors boost mTORC1 activity on lysosomes. **(B)** During low levels of amino acids: (1) Low intraluminal amino acids compromise inside out mediated activation of mTORC1. (2) The absence of arginine leads to a halt in leucine import via SLC38A9. (3) With no leucine in the lumen, the V-ATPase is unable to activate Ragulator and Rag GTPase heterodimers. (4) In addition, low cytosolic leucine prompts leucine export from the lysosome via PAT1, further depleting luminal leucine. (5) Decreased levels of cytosolic amino acids relieve sestrins and CASTOR1 from inhibition. (6) Free Sestrins and CASTOR1 can now bind and handicap GATOR2, freeing GATOR1. (7) GATOR1 is then recruited to the lysosome by the lysosome-associated KICSTOR, which further suppresses Ragulator/Rag GTPases. (8) Rag heterodimers take on GDP-RagA/B::GTP-RagC/D inhibitory conformation. (9) All these changes result in the release of mTORC1 from the lysosome and its suppression, thus initiating catabolic cellular programs.

To be activated, mTORC1 requires two major inputs: localization to lysosomes via Rag GTPases, followed by interaction with GTP-loaded Rheb GTPases (Figure 3). Focusing on Rag GTPases as the anchor for mTORC1 on lysosomes, Rag GTPase are themselves regulated by the Ragulator, a protein complex comprised of five subunits named p18, p14, MP1, C7orf59, and HBXIP (also known as LAMTOR-1 through -5, respectively) (Bar-Peled et al., 2012; Yonehara et al., 2017; Zhang T. et al., 2017; Rabanal-Ruiz and Korolchuk, 2018). Ragulator is anchored to the lysosome via the N-terminus of the p18 subunit, which possesses myristoylated and palmitoylated sites (Nada et al., 2009). Then, the p18 and MP1-p14 subunits tether the Rag GTPases to the lysosome (Zhang C.S. et al., 2017; Yonehara et al., 2017). The Rag family of small GTPases consists of two obligate heterodimer pairs, RagA or RagB bound to either Rag C or RagD (e.g., RagA/RagC) (Sekiguchi et al., 2001; Bar-Peled et al., 2012). When active, RagA or RabB are GTP-loaded and RagC or RagD are GDP-bound, while inactive Rag GTPase complexes have the converse nucleotide status (Sekiguchi et al., 2001; Bar-Peled et al., 2012; Tsun et al., 2013). Importantly, in response to specific amino acids, the Ragulator becomes a GEF towards RagA and RagB, loading these GTPases with GTP (Bar-Peled et al., 2012). It is in this form (GTP-RagA/B:GDP-RagC/D) that Rag GTPases are able to bind and anchor mTORC1 to the lysosomal surface (Kim et al., 2008; Sancak et al., 2008, 2010; Figure 4).

**FIGURE 5:**
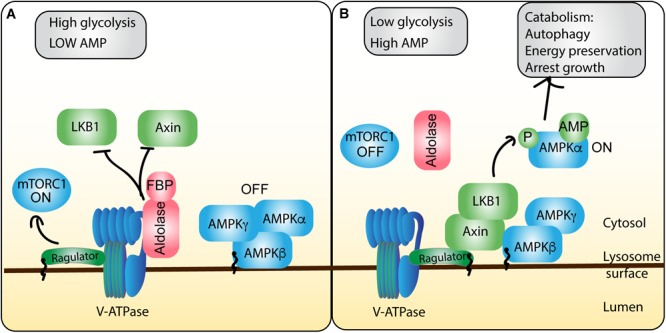
AMPK integration of energy stress shapes mTORC1 activity at the lysosome. ATP and glycolytic activity are sensed and integrated by the AMPK regulatory machinery on the lysosome, allowing for cross-talk to the mTORC1 pathway. **(A)** In conditions of energy sufficiency, there is high ATP levels relative to AMP, which permits ATP to compete with AMP for binding to AMPK, maintaining the inactive heterotrimeric AMPK complex. Moreover, high glycolytic activity produces abundant fructose-1,6-bisphosphate (FBP), which forms a complex with aldolase. The aldolase-FBP complex interacts with the V-ATPase to thwart recruitment of LKB1 and AXIN to the lysosome and protect V-ATPase-Ragulator association. Collectively, these events abate AMPK and stimulate mTORC1 activity. **(B)** During conditions of energy insufficiency, AMP concentrations rise, increasing the frequency of AMP-bound AMPK, priming AMPK for activation. Moreover, decreased glycolysis activity drops the levels of FBP and FBP-aldolase. This permits the formation of a complex between Ragulator, V-ATPase, Axin and LKB. Collectively, this allows LKB1 to phosphorylate and release the catalytic α subunit, allowing it to promote catabolic cellular programs, while preventing Ragulator from promoting mTORC1 function.

While we expand further on mTORC1 regulation and assembly on lysosomes below, we would be remiss if we did not also emphasize that amazingly, lysosomes are platforms for the assembly and cross-talk of other major metabolic circuits encoded by the AMPK and GSK3β signaling hubs. AMPK is a primary cellular sensor for energy stress and glucose levels, promoting catabolic programs in response to low energy levels (Carroll and Dunlop, 2017). During energy stress, the AXIN/Liver Kinase B 1(LKB1) complex associates, phosphorylates and promotes AMPK (Zhang et al., 2013; Figure 5). Strikingly, this appears to happen at the level of the lysosome as the scaffold protein AXIN binds LAMTOR1/p18, on the lysosomal surface, and then recruits the kinase LKB1 (Zhang C.S. et al., 2017). Concurrently, AXIN binds to and compromises the GEF capacity of Ragulator, impairing Rag GTPases, and leading to mTORC1 dissociation from the lysosomes (Zhang C.S. et al., 2017). In this way, AMPK and mTORC1 activities are coordinated with opposing effects. Finally, GSK3β is a kinase with 500 putative substrates and seemingly contradictory functions that may be dictated by GSK3β localization (Beurel et al., 2015); for example, GSK3β localization to lysosomes may promote cell survival and growth, while its localization to the nucleus may promote cell death functions (Bautista et al., 2018). Indeed, its localization to the lysosomes may permit GSK3 to phosphorylate Raptor on Ser859, in response to amino acids, promoting increased Raptor-mTORC1 interactions (Stretton et al., 2015). Overall, lysosomes have emerged as a major signaling platform to assemble critical molecular circuits that coordinate decisions on cell growth, division, and survival. We next elaborate in greater detail about these environmental sensing mechanisms present on lysosomes.

### Growth Factor Signaling Is Sensed at the Lysosomal Surface

Growth factor, mitogen, hormone and cytokine signaling initiate anabolic cellular programs to engage cell division, stress response, or inflammation. Many of these signal transduction events communicate to the lysosomal surface to coordinate mTORC1 through the Rheb GTPase that upregulates mTORC1 kinase activity in parallel to Rag GTPase-mediated mTORC1 recruitment to lysosomes (Figure 3). Rheb GTPases are themselves inactivated by the lysosome-localized Tuberous Sclerosis Complex (TSC), composed of TSC1, TSC2 and TBC1D7 subunits and forming a Rheb GTPase-activating protein (GAP) (Inoki et al., 2002; Dibble et al., 2012; Menon et al., 2014). Specifically, receptor signaling stimulates PI 3-kinases to generate PIP_3_ at the plasma membrane (Menon et al., 2014; Zheng et al., 2014; Dibble and Cantley, 2015). PIP_3_ then recruits and stimulates the kinase Akt, which is then able to directly phosphorylate S939, S981, S1130, S1132, and T1462 on TSC2 (Inoki et al., 2002; Potter et al., 2002; Roux et al., 2004; Huang and Manning, 2008; Dibble and Cantley, 2015). This dissociates the TSC complex from the lysosomes, alleviating the GAP activity on Rheb GTPase (Inoki et al., 2002; Dibble et al., 2012; Menon et al., 2014). GTP-bound Rheb can then bind and activate mTORC1 via the mTOR subunit, if mTORC1 is localized to lysosomes (Figure 3, 4; Garami et al., 2003; Long et al., 2005; Zheng et al., 2014). While the PIP_3_-Akt pathway is the best characterized modulator of the TSC complex, additional pathways can complement, strengthen or counteract the effects of Akt. First, various receptors stimulate kinases ERK1/2, which then acts on the RSK that then phosphorylates and represses TSC2 (Roux et al., 2004). Second, the ubiquitination status of Rheb governs its association with the TSC complex (Deng et al., 2018). In particular, the lysosome-anchored E3 ligase RNF152 ubiquitinates Rheb, resulting in its association with TSC and subsequent inactivation (Deng et al., 2018; Figure 3). However, in response to EGF, Rheb is deubiquitinated through Akt-mediated phosphorylation of the deubiquitinase USP4, which enables Rheb to dissociate from TSC, promoting mTORC1. Overall, the TSC and Rheb are subjected to diverse inputs emanating from receptor-signaling that shapes mTORC1 activity on the lysosomal surface. We end this section by noting that the Rheb sub-cellular localization is currently controversial. While mTORC1 and TSCs are clearly in the lysosome, over-expressed (Jiang and Vogt, 2008; Hanker et al., 2010) and endogenous Rheb (Hao et al., 2017) appear to localize to ER and/or the Golgi, with this distribution able to regulate mTORC1. One possibility is that ER/Golgi-localized Rheb is able to modulate mTORC1 or be governed by TSCs through membrane-contact sites between lysosomes and the ER/Golgi (Hao et al., 2017).

### Sensing the Levels of Amino Acids in the Lysosome Lumen

For GTP-bound Rheb to associate and stimulate mTORC1 kinase activity, mTORC1 must be localized to lysosomes, a step that is controlled by the availability of specific amino acids, particularly leucine, arginine, glutamine and methionine (Lim and Zoncu, 2016; Carroll and Dunlop, 2017). The lysosome employs both cytosolic and “inside-out” mechanisms to sense and respond to amino acid concentrations that interface with the machinery that governs mTORC1 activity (Figure 4). We start this discussion with inside-out signaling, which relies on intraluminal sensing of amino acids.

First, lysosomal arginine is sensed by the sodium coupled amino acid transporter SLC38A9 (Jung et al., 2015; Rebsamen and Superti-Furga, 2016). Specifically, arginine-bound SLC38A9 undergoes conformational changes that permit interactions with the Ragulator complex enabling mTORC1 activation (Wang et al., 2015). Additionally, arginine binding allows SLC38A9 to interact with and stimulate GTP-loading onto the RagA GTPase, thus acting as a GEF (Shen and Sabatini, 2018). GTP-RagA is then released from SLC38A9 to bind mTORC1, which also frees SLC38A9 for additional rounds of Rag stimulation on the lysosomal surface (Shen and Sabatini, 2018). Interestingly, high arginine levels within lysosomes stimulates SLC38A9 to transport intraluminal amino acids like leucine into the cytosol, generating a mechanism that allows intraluminal amino acid sensors to cross-talk with sensors that detect cytosolic amino acids, discussed below (Wyant et al., 2017).

Second, intraluminal leucine itself is a potent activator of mTORC1 lysosomal recruitment, albeit through different means. There are multiple mechanisms that help stockpile leucine within lysosomes. For one, as leucine concentration rises in nutrient-rich conditions, FLCN helps sequester leucine within the lysosome by impairing the lysosomal PAT1; conversely, under starvation conditions, PAT1 facilitates efflux of leucine from the lysosome (Wu et al., 2016). On the other hand, leucine from the cytosol can be imported into and amass within the lysosome; this happens because the lysosomal membrane protein LAPTM4b retains the leucine transporter LAT1-4F2hc on lysosomes, which then imports leucine in exchange for non-essential amino acids (Milkereit et al., 2015). Collectively, high intraluminal leucine concentration promotes ATP hydrolysis by the V-ATPase, enabling interactions with and stimulating the Ragulator-Rag complex to support mTORC1 recruitment to the lysosome (Zoncu et al., 2011; Milkereit et al., 2015). V-ATPase assembly and activity is further boosted by the lysosomal protein TMEM55B, which recruits the V_1_ peripheral membrane subcomplex to lysosomes in response to leucine (Hashimoto et al., 2018). The corollary of all these mechanisms is stimulation of Ragulator, Rag GTPases and ultimately mTORC1.

### Lysosome Sensing of Cytosolic Amino Acid Levels

Arginine and leucine in the cytosol are also both sensed by and regulate the cytosolic GATOR1-GATOR2 complexes to modulate mTORC1 recruitment to lysosomes (Bar-Peled et al., 2013; Peng et al., 2017; Figure 4). The GATOR1 complex is recruited to lysosomes by the KICSTOR complex, where it then acts as a GAP for RagA/B GTPases, antagonizing the Ragulator complex, and thus, displacing mTORC1 from lysosomes (Bar-Peled et al., 2013; Wolfson et al., 2017). In the presence of amino acids, the GATOR2 complex seems to associate and impair GATOR1, eliciting GTP-loading on RagA/B and activating mTORC1 (Bar-Peled et al., 2013). The balance between GATOR1-GATOR2 outputs depend on several cytoplasmic protein complexes that sense arginine and leucine (and other metabolites). First, leucine binds to sestrins to modulate GATOR2; specifically, high leucine levels increases the proportion of leucine-bound sestrins, which are inhibited and unable to block GATOR2, which is then able to impede GATOR1. Conversely, low cytosolic leucine concentrations, increases the number of leucine-free sestrins, which bind and block GATOR2, permitting GATOR1 to down-regulate GTP-bound RagA/B (Parmigiani et al., 2014; Kimball et al., 2016; Wolfson et al., 2016; Peng et al., 2017). Secondly, arginine binds and blocks the CASTOR1, another inhibitor of GATOR2. Thus, at high-levels of arginine, CASTOR1 is bound and inhibited by arginine, releasing GATOR2 to block GATOR1. Conversely, at low-levels of arginine, more CASTOR1 is free to block GATOR2, releasing GATOR1 to block RagA/B-mediated recruitment of mTORC1 (Chantranupong et al., 2016; Saxton et al., 2016).

Interestingly, leucine and arginine can regulate mTORC1 activity using additional cytoplasmic mechanisms. The leucyl-tRNA synthetase is another cytoplasmic leucine sensor that potentiates Rag GTPases (Han et al., 2012; Lee M. et al., 2018). Specifically, leucyl-tRNA synthetase was discovered to be a GAP for the RagD GTPase (Lee M. et al., 2018). RagD^GDP^ promotes increased affinity for Ragulator interactions with the RagB/D heterodimer, loading GTP onto RagB to foster mTORC1 recruitment (Lee M. et al., 2018). Similarly, arginine compromises TSC-Rheb interactions by displacing TSC complex into the cytosol (Carroll et al., 2016). We note that this brief overview is not exhaustive of inside-out and cytoplasmic amino acid sensing that converge on the lysosome and that additional mechanisms like glutamine sensing exist (Nicklin et al., 2009). Overall, a key challenge in the field is to understand how all these individual signals are integrated to modulate and balance mTORC1 activity to best serve the needs of the cell.

### The Lysosome as a Sensor for Cellular Energy Status

AMP-activated protein kinase is a heterotrimeric protein composed of the α kinase subunit, and the regulatory β and γ subunits that monitors the AMP:ATP ratio (Hardie, 2014; Carroll and Dunlop, 2017; Lin and Hardie, 2018; Rabanal-Ruiz and Korolchuk, 2018; Figure 5). ATP-bound AMPK maintains all three subunits together in an inactive pool. However, as energy levels drop, AMP levels increase, leading to increased AMP binding to regulatory subunits, which frees the catalytic subunit to act on numerous targets that stimulate catabolism and repress anabolic processes (Carroll and Dunlop, 2017; Lin and Hardie, 2018). Remarkably, energy and sensing of stresses like hypoxia and DNA damage by AMPK-based circuits occurs at the lysosome surface.

Low energy states permit AXIN to nucleate a complex between LKB1, AMPK, the Ragulator p18 subunit, and the V-ATPase, wherein V-ATPase promotes AXIN-Ragulator interactions, (Zhang et al., 2013; Zhang C.S. et al., 2017; Figure 5). Myristoylation of the β subunit of AMPK also helps localize AMPK to lysosomal membranes (Oakhill et al., 2010; Zhang C.S. et al., 2017). This assembly ultimately allows LKB1 to phosphorylate AMPK on Thr172 of the α subunit, releasing the catalytic subunit, which can now act to suppress anabolism and promote catabolism (Davies et al., 1995; Crute et al., 1998; Hawley et al., 2003; Gowans et al., 2013). In part, this is accomplished by AMPK-dependent phosphorylation of TSC, which promotes its localization to lysosomes to repress Rheb GTPases and mTORC1 (Inoki et al., 2003, 2006).

Interestingly, this AMPK-regulatory hub was recently discovered to be regulated by glycolytic byproducts and enzymes at the lysosome; specifically, low FBP, a product of glycolysis, promotes AMPK activation and subsequent mTORC1 inactivation (Zhang C.S. et al., 2017). These authors discovered that aldolase, an activator of pyruvate kinase acts as a sensor for FBP, which upon binding, localizes to the lysosome where it interacts with v-ATPase, disrupting AXIN/LKB1 interactions with the V-ATPase-Ragulator complex and thus permits mTORC1 activation (Figure 5). However, under low glucose conditions, FBP concentrations drop, and aldolase cannot bind FBP. Consequently, this compromises aldolase-FBP binding to V-ATPase and promotes the assembly of the AXIN/LKB1-Ragulator-V-ATPase complex, which (i) promotes AMPK phosphorylation and (ii) subsequent mTORC1 dissociation from the lysosome by disrupting Ragulator and Rag GTPases (Zhang C.S. et al., 2017). Interestingly the authors suggest that aldolase acts to sense falling glucose levels, preemptively recruiting AMPK to lysosomes, before changes in the cellular AMP:ATP ratio occur, that can directly be sensed by AMPK.

AMP-activated protein kinase is also involved in regulating metabolism in response to other stresses like DNA damage. For example, the tumor suppressor protein p53 is capable of inducing expression of Sestrins, which in turn phosphorylate the α subunit of AMPK (Budanov and Karin, 2008; Budanov, 2011; Lee et al., 2012). Consequently, activated AMPK is able to phosphorylate TSC2, promoting GAP activity towards Rheb GTPase (Budanov and Karin, 2008). AMPK can also promote hyperphosphorylation of TFEB (more about TFEB below), resulting in greater nuclear shuttling of TFEB and thus promotion of catabolic cellular programs (Young et al., 2016). Overall, lysosome serves to integrate energy and stress conditions like DNA damage by interfacing with AMPK to promote catabolic and stress-resolution programs.

### The Lysosome Facilitates Immune Sensing

**FIGURE 6:**
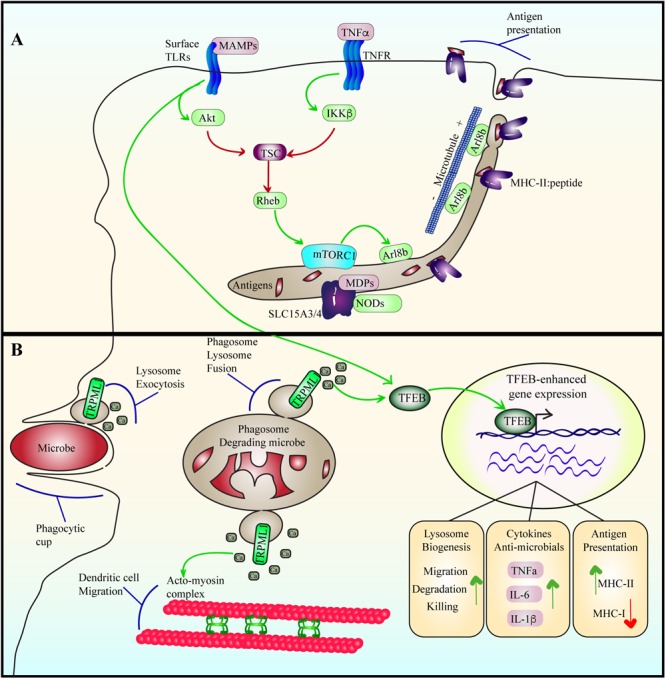
Lysosome adaptation and remodeling during immune responses. **(A)** Cytokine and TLR signaling stimulate mTORC1, likely by suppressing TSC using Akt and IKKβ. Among other functions, LPS-TLR4 activation of mTORC1 may stimulate the levels of Arl8b on lysosomes, which coordinates with kinesin-1 to drive lysosome tubulation and extension towards the cell periphery. This may then aid in antigen presentation in dendritic cells. Tubulation may also aid in increasing the surface area-to-volume ratio of lysosomes to favor antigen processing and export or other functions. For example, the SLC15A3/4 transporters export muramyl dipeptides originating from gram-positive bacteria to induce NOD signaling. **(B)** During phagocytosis, microbes are engulfed and sequestered into phagosomes. Engulfment is aided by lysosome exocytosis triggered by release of lysosomal Ca^2+^ via TRPML1. Phagosomes then mature by fusing with lysosomes to digest the particle. Phagosome-lysosome fusion is catalyzed by TRPML1-mediated Ca^2+^ release from lysosomes as well. TFEB is activated by LPS-TLR4 signaling in a delayed fashion or by phagocytosis of bacteria. During phagosome maturation, TRPML1-mediated Ca^2+^ elicits nuclear entry of TFEB to upregulate lysosome gene expression, enhancing the degradative and killing capacity of macrophages. In dendritic cells, TFEB activation enhances TRPML1 expression, which aids in intralysosomal Ca^2+^ release that coordinates the acto-myosin machinery to facilitate dendritic cell migration.

While the lysosome can sense nutrient and energy levels to shape cellular metabolism, it is also capable of detecting the onset of infection and help facilitate immune responses against emerging pathogen threats (Figure 6). This is partly encoded by effects on the lysosome-localized TSC via cytokine signaling. For example, TNFα, a pro-inflammatory cytokine, leads to phosphorylation of S487 and S511 and inhibition of the TSC1 subunit via the kinase IKKβ (Lee et al., 2007). Consequently, mTORC1 activity is stimulated. Similarly, Toll-like receptors (TLRs) also interface with lysosomes to modulate inflammation, metabolism and antigen processing. TLRs recognize microbe associated molecular patterns to elicit cytokine and chemokine expression to coordinate an immune response; for example, plasma membrane TLR4 and TLR2 recognize bacterial LPS and peptidoglycans, respectively, while endosome/lysosome-localized TLR7 and TLR9 sense viral and bacterial nucleic acids (Takeda et al., 2003; Kawai and Akira, 2010; Tohmé and Manoury, 2014; Dolasia et al., 2018). With respect to LPS-TLR4, mTORC1 undergoes activation, possibly through stimulation of phosphatidylinositol 3-kinase and Akt (Luo et al., 2014; Saric et al., 2016), and/or as shown recently, TLR3 and TLR4 induce TBK1-mediated phosphorylation and activation of mTORC1 (Bodur et al., 2018). Regardless, LPS exposure to macrophages and dendritic cells use mTORC1 to remodel the lysosomal system from a vesicular population to a tubular network that may promote antigen presentation (Vyas et al., 2007; Saric et al., 2016; Hipolito et al., 2018).

Internal TLRs require proteolytic cleavage by lysosomal cathepsins in order to initiate signal transduction events in response to ligand binding (Ewald et al., 2008; Park et al., 2008; Garcia-Cattaneo et al., 2012). Proteolytic cleavage of TLRs is believed to be a mechanism in which TLRs can discriminate from self and foreign nucleic acids, wherein only cleaved TLRs can activate their adaptor proteins and downstream cytokine expression (Ewald et al., 2008; Park et al., 2008). For example, TLR 3, 7, and 9 are transported from the ER to lysosomes, where they are cleaved by asparagine endopeptidase and several cathepsins (Ewald et al., 2008, 2011; Park et al., 2008; Garcia-Cattaneo et al., 2012; Tohmé and Manoury, 2014). TLR9 actually exists in lysosomes as both an intact receptor and as a cleaved C-terminal fragment, wherein the ectodomain has been cleaved (Ewald et al., 2008; Park et al., 2008). Both have ligand binding ability, but only cleaved TLR9 can initiate immune signaling; this effectively restricts activation to lysosomal compartments (Ewald et al., 2008; Park et al., 2008). Akin to inside-out amino acid sensing, we see here a unique example of inside-out immune sensing within a lysosomal compartment, which allows for downstream immune responses to initiate.

### Sensing Membrane Damage

Damage to the plasma membrane and to internal membrane compartments can be sensed by lysosomes to initiate membrane repair or clearance mechanisms. For instance, the plasma membrane can suffer tears caused by mechanical stresses during cell migration (Mayer et al., 2004; Mellgren, 2011), or through therapeutic applications like ultrasound-activated microbubble sonoporation that is being explored to locally deliver drugs to tumors (Mariglia et al., 2018). Given the high concentration of extracellular Ca^2+^ relative to cytosolic Ca^2+^, there is a rapid and local influx of Ca^2+^ into the cytosol during plasma membrane damage (Andrews, 2000; Cheng et al., 2015; Davenport et al., 2016). This is ultimately sensed by lysosomes near the plasma membrane expressing Rab3a GTPase and Synaptogamin VII, a lysosomal transmembrane protein that binds to Ca^2+^ to promote lysosome fusion (Martinez et al., 2000; Reddy et al., 2001; Jaiswal et al., 2002; Encarnação et al., 2016). Synaptogamin VII on lysosomes undergoes conformational changes that enable it to interact with VAMP-7 on lysosomes and SNAP-23 and syntaxin-4 on the plasma membrane to induce fusion (Rao et al., 2004). As described below, lysosome exocytosis then aids in repairing plasma membrane lesions (Reddy et al., 2001; Jaiswal et al., 2002; Rao et al., 2004; Cheng et al., 2015; Encarnação et al., 2016).

Organelles like endosomes, lysosomes and phagosomes can also sustain damage; for example, internalization of toxins like *Anthrax lethal toxin* (Bernheimer and Schwartz, 1964; Averette et al., 2009), or microbe escape from phagosomes, as accomplished by *Listeria* (Hara et al., 2007; Smith and May, 2013). Detection of damage usually proceeds by exposing danger-associated molecular patterns like luminal glycoproteins or glycosylated luminal domains of transmembrane proteins, which are normally inaccessible to cytosolic receptors (Thurston et al., 2012; Sundblad et al., 2013; Yoshida et al., 2017). These receptors include proteins like the cytosolic galectins, lectins that recognize and bind the oligosaccharide moieties like β-galactosides (Thurston et al., 2012; Maejima et al., 2013; Sundblad et al., 2013; Chauhan et al., 2016). Galectins then interact with TRIM-family proteins, which act as autophagic receptors that lead to ubiquitylation of damaged organelles (Chauhan et al., 2016; Kumar et al., 2017). Galectins were also reported to locally displace mTORC1 and stimulate AMPK from the damaged lysosome, aiding in targeted autophagy (Jia et al., 2018). In particular, lysosomal membrane damage stimulates galectin -8 and 9 (Gal8, Gal9) (Jia et al., 2018). Gal8 labels damaged lysosomes by interacting with exposed glycosylated residues on SLC38A9, which then inhibits Ragulator and dissociates mTORC1 from the damaged lysosome (Jia et al., 2018, 2019) In contrast, Gal9 activates TAK1, a kinase that phosphorylates Thr172 and stimulates AMPK (Jia et al., 2018, 2019). Overall, the lysosome serves as a platform for signaling complexes to initiate membrane repair and clearance responses. We will explore the actual process of clearance below.

## The Lysosome Transduces and Mediates Cellular Outcomes

The following sections focus on outcomes generated by lysosomal-based sensors and integrators discussed above, particularly mTORC1, AMPK and immune receptors. We dedicate a discussion to autophagy initiation, ALR, lysosome adaptation, antigen presentation and membrane repair. By no means is the following discussion exhaustive of physiological outcomes dictated by lysosome-based signaling circuits, which can also include mitochondrial adaptation and regulation (Daniele and Schiaffino, 2016; Wong et al., 2018), adaptation to different carbon sources (Han and Emr, 2011), cholesterol metabolism (Castellano et al., 2017; Pfeffer, 2019), reactive oxygen species signaling (Zhang et al., 2016), and hypoxic response (Lai et al., 2016).

### Initiation of Autophagy

Autophagy is one of the key outcomes elicited by mTORC1 suppression and AMPK activation caused by amino acid and/or energy depletion, which aims to recycle and recover nutrients to help maintain homeostasis (Parzych and Klionsky, 2014; Bento et al., 2016; Zhao and Zhang, 2019). The kinase ULK1 is one of the most upstream initiators of autophagy that induces phagophore formation and elongation by stimulating the Vps34 lipid kinase complex II to generate PI(3)P synthesis on phagophores (Russell et al., 2013; Parzych and Klionsky, 2014; Bento et al., 2016). However, in nutrient rich conditions, mTORC1 phosphorylates ULK1 on Ser757 and its partner, Atg13, to impede ULK1 action (Ganley et al., 2009; Rabanal-Ruiz and Korolchuk, 2018). Furthermore, mTORC1 phosphorylates and inactivates, Atg14, a protein component of the Vps34 lipid kinase complex, suppressing phagophore growth (Yuan et al., 2013). Conversely, during starvation, mTORC1 is inactivated and dissociates from lysosomes, releasing the brake imposed on ULK1 and the VPS34 complex, which can now initiate and promote phagophore elongation (Yuan et al., 2013; Park et al., 2018; Zhao and Zhang, 2019).

AMP-activated protein kinase also facilitates autophagy initiation by directly and indirectly stimulating ULK1 activation. First, AMPK represses mTORC1 activity through phosphorylation of TSC and Raptor, a component of mTORC1, which allows for 14-3-3 protein binding and mTORC1 inactivation (Gwinn et al., 2008; Kim and Guan, 2011; Kim et al., 2011). As a result, mTORC1 dissociates from the lysosomal membrane, reprieving ULK activity. Second, AMPK binds to and phosphorylates Ser317 and Ser777 of ULK1 under starvation conditions to directly stimulate ULK1, thus buttressing autophagy initiation (Kim and Guan, 2011; Martini-Stoica et al., 2016). Ultimately, autophagy culminates in the enclosure of target cytoplasmic material within a double-bilayer autophagosome, which fuses with lysosomes to form autolysosomes. The enclosed cargo is digested, free amino acid concentration increases, which ultimately reactivates mTORC1 to suppress autophagy. For deeper discussion of autophagy regulation and function, the reader is directed to the following excellent reviews (Bento et al., 2016; Noda, 2017; Rabanal-Ruiz and Korolchuk, 2018; Zhao and Zhang, 2019).

### Autophagy Lysosome Reformation

Cargo digestion within autolysosomes releases a bolus of essential amino acids and energy sources that reactivate mTORC1 using inside-out signaling pathways discussed above (Rabanal-Ruiz and Korolchuk, 2018). Strikingly, this mTORC1 reactivation does not simply drive anabolic reactions like protein synthesis, but orchestrates a highly localized process of reforming lysosomes from spent autolysosomes by ALR (Yu et al., 2010). During ALR, lysosome-like tubules emerge from autolysosomes to form proto-lysosomes that then mature into lysosomes to enable autophagic flux (Yu et al., 2010; Chen and Yu, 2017). The molecular mechanisms driving ALR are beginning to emerge but seems to require localized, waves of phosphoinositide signaling. First, mTORC1 catalyzes synthesis of PtdIns(4,5)P_2_ on mature autolysosomes via PIP5K1B, which then nucleates clathrin-AP2 complexes that help deform the membrane (Rong et al., 2012). This bud is then deformed into a tubule in part through recruitment of KIF5B, a kinesin motor that binds to PtdIns(4,5)P_2_ (Du et al., 2016). Tubules are then scissioned at the distal end by a second round of PtdIns(4,5)P_2_ driven by PIP5K1A, which assembles clathrin-dynamin complex to release proto-lysosomes (Rong et al., 2012). Interestingly, lysosomal PtdIns(4)P and PtdIns(3)P also coordinate lysosome tubulation and recycling processes (Sridhar et al., 2013; Munson et al., 2015; Saffi and Botelho, 2019). An interesting challenge will be to understand if ALR is a monolithic process or a heterogeneous process regulated by distinct mechanisms and producing distinct lysosome recycling compartments.

### Transcriptional Regulation of Lysosome Function in Response to Starvation

Lysosomes were once thought to be static organelles, but we now appreciate that cells evolved mechanisms to adapt lysosomes in response to a variety of stresses (Hipolito et al., 2018). In part, lysosomal adaptation occurs at the transcriptional level by engaging various transcription regulators including TFEB, TFE3, p53, FOXO, ZKSCAN3, and PPARα (Sardiello et al., 2009; Chauhan et al., 2013; Webb and Brunet, 2014; Simon et al., 2017; Brady et al., 2018; Hesketh et al., 2018). Of these, engagement of TFEB and TFE3 is the best characterized mechanism of lysosome adaptation in retort to starvation. TFEB and TFE3 both bind to promoter sequences containing the CLEAR sequence element, forming the CLEAR gene network. This gene network is enriched in genes encoding lysosome, endosome, and autophagy proteins; thus, stress-induced activation of TFEB/TFE3 can adapt and scale-up the activity of the endo-lysosomal system by driving lysosome biogenesis and autophagic flux (Palmieri et al., 2011; Settembre and Ballabio, 2011; Settembre et al., 2011; Martina et al., 2014). Interestingly, lysosomal adaptation is potentiated by a self-induced positive feedback loop driven by TFEB, whose gene also carries a CLEAR element, enabling TFEB to drive its own expression (Settembre et al., 2013). As is the theme of this review, control of the nucleo-cytoplasmic shuttling of TFEB/TFE3 occurs at the level of lysosomes.

Transcription factor EB activity is modulated by various post-translational modifications that toggle TFEB between the cytoplasm (inactive), lysosomal surface, and nuclear entry and chromatin association. To date, >20 phosphorylation, acetylation and sumoylation sites and several kinases responsible for phosphorylation of these sites have been characterized (Puertollano et al., 2018). Of these, the best characterized regulator of TFEB/TFE3 is mTORC1. In nutrient rich conditions, mTORC1 on lysosomes phosphorylates TFEB on Ser122 and Ser211, the latter forming a docking site to bind YWHA/14-3-3 proteins that mask TFEB’s nuclear localization sequence (Figure 2; Martina et al., 2012; Roczniak-Ferguson et al., 2012; Settembre et al., 2012; Vega-Rubin-de-Celis et al., 2017). We should note that mTORC1 has also been proposed to phosphorylate several serines on the C-terminal of TFEB to stimulate TFEB– this discrepancy remains unresolved (Peña-Llopis et al., 2011; Puertollano et al., 2018). Dephosphorylation of TFEB is partly mediated by calcineurin, a phosphatase activated by the efflux of lysosomal Ca^2+^ through MCOLN1 triggered during starvation (Medina et al., 2015; Zhang et al., 2016).

A recent development in TFEB regulation shows the unexpected complexity of this system. Work by Sha et al. showed that the STUB1, an E3 ubiquitin ligase, preferentially ubiquitylates phosphorylated and inactive TFEB, leading to its degradation by the proteasome, while dephosphorylated and active TFEB was spared this fate and accumulated in nucleus; consequently, there is increased heterodimerization of inactive and active TFEB isoforms in STUB1-deficient cells, compromising nuclear localization of the active form even during starvation (Sha et al., 2017). Thus, STUB1 promotes TFEB activity by preventing heterodimerization of inactive and active TFEB by targeting inactive TFEB to ubiquitin-proteasome degradation pathway. For additional focused discussion on TFEB, we point the reader to the following recent reviews (Sardiello, 2016; Brady et al., 2018; Puertollano et al., 2018; Yang et al., 2018).

While TFEB and TFE3 scale up lysosomal function and adaptation, ZKSCAN3 does the opposite. ZKSCAN3 is a zinc finger transcription regulator that is widely regarded as a master repressor of autophagy. Using ChIP-seq analysis, ZKSCAN3 was shown to bind to promoter regions of autophagic and lysosomal genes and repress their expression (Chauhan et al., 2013). Complementing this observation, disruption of ZKSCAN3 induced autophagy and increased lysosomal biogenesis (Chauhan et al., 2013). ZKSCAN3 activity is shaped by the nutrient status of the cell and associated mTORC1 activity as well. However, in contrast to TFEB, starvation and mTORC1 inhibition causes ZKSCAN3 to shuttle out of the nucleus and into the cytosol (Chauhan et al., 2013; Lim and Zoncu, 2016). Ultimately, what we see is a regulatory network that involves lysosomal nutrient sensing which signals to protein complexes on the lysosomal membrane, in this case mTORC1, to repress or promote the expression of genes needed for cellular and lysosome adaptation to stress.

### Metabolic and Survival Decisions: GSK3 Weighs in

The GSK3 is another lysosomal transducer that coordinates changes to metabolism, autophagy, cell cycle checkpoints and stress resolution by targeting up to 500 putative targets (Linding et al., 2007; Beurel et al., 2015). The localization of GSK3 to cytosol, plasma membrane, nucleus and lysosomes is thought to enable specificity in a context-dependent manner. For example, recent work revealed that lysosomal GSK3 is modulated by mTORC1 to govern GSK3 activity and localization (Bautista et al., 2018). Specifically, active mTORC1 retains GSK3 in the cytoplasm and/or on lysosomes in a manner that required Rab7-mediated trafficking. On the other hand, mTORC1 inactivation or disruption of Rab7 caused GSK3 to translocate to the nucleus, where it was able to phosphorylate and degrade the transcription factors like c-Myc and SNAIL, which promote cell proliferation and anabolic programs (Miller et al., 2012; Bautista et al., 2018). Thus, loss of mTORC1 function and lysosomal trafficking enables GSK3 translocation to the nucleus, ceasing cell growth programs and supporting catabolic programs such as autophagy. Remarkably, cytoplasmic GSK3 can have the opposite effect by phosphorylating Raptor on Ser859 in response to amino acids (Stretton et al., 2015). This phosphorylation event leads to stronger interactions between Raptor and mTOR, aiding in mTORC1-mediated suppression of downstream actors such as TFEB and ULK1 (Stretton et al., 2015). Thus, mTORC1 and GSK3 form a complex bi-directional circuit, whose outcome depends on their localization within cells. GSK3 itself can also interface and adapt lysosome function by acting directly on TFEB (Parr et al., 2012). For example, inhibition of GS3K leads to reduced levels of amyloid precursor protein (APP) in models of Alzheimer’s by stimulating TFEB nuclear entry and promoting autophagy (Parr et al., 2012). Interestingly, these models of Alzheimer’s display higher GSK3 activity, which may repress autophagy and stress resolution pathways leading to neuronal damage (Parr et al., 2012). Altogether, we can see that different transducers on the lysosome interact in context specific ways to facilitate relevant downstream events including cell survival and growth.

### Lysosomes in Phagocytosis, Phagosome Maturation and Cytokine Response

During phagocytosis, the plasma membrane of phagocytes is deformed to entrap microbes and other unwanted particulates, ultimately sequestering these within a phagosome. Phagosomes eventually fuse with lysosomes to degrade the offending microbe/particulate (Gray and Botelho, 2017). As with the metabolic responses above, the role of lysosomes in immunity goes beyond that of degradation; lysosomes help immune cells sense, respond and adapt to infections and other immune stresses. First, phagocytosis of large antibody-coated particles requires focal exocytosis of endomembranes to help grow the phagocytic cup and pre-empt cell shrinkage due to the large membrane intake (Figure 6). While recycling endosomes and even the ER were suggested to undergo exocytosis (Bajno et al., 2000; Gagnon et al., 2002), lysosomes were also observed to undergo secretion to complete phagocytosis (Samie et al., 2013; Haka et al., 2016). Exocytosis of lysosomes onto phagocytic cups required efflux of lysosomal Ca^2+^ via TRPML1 to activate synaptotgamin VII, which then promotes lysosomal exocytosis (Andrews, 2000; Czibener et al., 2006; Samie et al., 2013). Perhaps, co-opting this system for their own survival, uropathogenic *E. coli* were shown to neutralize the lysosome luminal pH, which activates TRPML3 to release lysosomal Ca^2+^, triggering lysosomal exocytosis and ejection of uropathogenic *E. coli*, henceforth avoiding digestion within lysosomes (Miao et al., 2015). Second, in addition to lysosome exocytosis, TRPML also mediates fusion of lysosomes docked onto phagosomes in macrophages and neutrophils by releasing intra-lysosomal Ca^2+^ (Dayam et al., 2015). Indeed, supplementation of Ca^2+^ was sufficient to rescue fusion of frustrated, docked phagosome-lysosomes in TRPML1-silenced cells (Dayam et al., 2015). Finally, TRPML1-mediated efflux of lysosomal Ca^2+^ during phagosome maturation was also responsible for activating TFEB in mammalian macrophages, enhancing the degradative and bactericidal properties in response to phagocytosis (Gray et al., 2016). Overall, lysosome machinery is able to aid in phagocytosis, phagosome maturation and adapt lysosomes to phagocytosis.

The role of TFEB in immunity is evolutionary conserved and potentially represents an ancient function of TFEB. Indeed, *Caenorhabditis elegans* activates HLH30, its TFEB ortholog, in response to *Staphylococcus aureus* infection to promote the expression of host defense genes as well as genes related to autophagy and lysosome function (Visvikis et al., 2014). Deletion of HLH30 makes *C. elegans* prone to infection and death (Visvikis et al., 2014). As with Fcγ receptor-mediated phagocytosis (Gray et al., 2016), *Salmonella* and *S. aureus* infection in murine macrophages activated TFEB, which also promoted the expression of several immune-protective genes, cytokines and chemokines such as IL1β, IL-6, TNFα, and CCL5 (Visvikis et al., 2014; Najibi et al., 2016). Interestingly, macrophage exposure to LPS alone was sufficient to activate TFEB and TFE3, promoting lysosomal, autophagy and various cytokine and chemokines *in vitro*, an effect abrogated in single and double knockout cells of TFEB and/or TFE3 (Pastore et al., 2016). Importantly, mice defective in TFEB and/or TFE3 also displayed altered cytokine profiles in response to LPS injection (Pastore et al., 2016). However, while phagocytosis with IgG-coated particles or whole bacteria activates TFEB/TFE3 within 1 h of uptake, LPS requires about 6 h to stimulate these proteins, suggesting an indirect role in activation (Gray et al., 2016; Pastore et al., 2016). Moreover, both treatments activate mTORC1, suggesting that TFEB/TFE3 are controlled through pathways that bypass mTORC1-mediated repression of TFEB/TFE3 (Gray et al., 2016; Pastore et al., 2016). On the other hand, reduction in mTORC1 activity caused by deletion of Lamtor1 or Raptor in macrophages elevated cytokine production in a TFEB-dependent manner in response to LPS (Hayama et al., 2018). Together, these findings suggest that TFEB modulation of immune function is subject to multiple inputs, including mTORC1-dependent and independent; importantly, the mechanisms that trump mTORC1-mediated repression of TFEB remains obscure.

### Lysosome Adaptation, Reorganization and Antigen Presentation

Dendritic cells control degradation of microbes and antigens to preserve peptides that are then loaded onto the MHC, followed by secretion and presentation to T cells. Specifically, MHC-II complex is found in and loaded with peptides processed within lysosomes, followed by exocytosis for presentation to CD4^+^ T helper cells (Mantegazza et al., 2013; Stern and Santambrogio, 2016). In contrast, MHC-I requires cross-presentation, whereby antigens from within lysosomes and phagosomes are exported into the ER, loaded onto MHC-I and then secreted for presentation to cytotoxic CD8^+^ T cells (Gil-Torregrosa et al., 2004; Cruz et al., 2017). The choice between MHC-I vs. MHC-II antigen presentation may depend on modulation of lysosome properties.

First, MHC-II::peptide presentation may occur through tubular lysosome intermediates. Activation of macrophages and dendritic cells causes a profound transformation of vesicular lysosomes into a tubular lysosome network through a process that remains poorly understood (Hipolito et al., 2018; Perrin et al., 2019). Interestingly, tubular lysosomes were observed to grow outwards to the cell periphery and towards immune synapses formed between dendritic cells and T cells (Boes et al., 2002; Chow et al., 2002). Consequently, lysosome tubulation was proposed to deliver MHC-II::peptides to the cell surface for presentation. This is consistent with work showing that the lysosomal Arl8b GTPase is required for MHC-II-mediated antigen presentation, which anchors kinesin to lysosomes (Garg et al., 2011; Michelet et al., 2015; Pu et al., 2015). Work from our lab also suggests that LPS-mediated mTOR activation stimulates Arl8b loading onto lysosomes, promoting tubulation and secretion of MHC-II (Saric et al., 2016). Lysosome tubulation may enrich lysosome transport intermediates in MHC-II::peptides relative to luminal enzymes by increasing the membrane area to volume ratio. Similarly, tubulation may aid the lysosomal peptide transporters SLC15A3 and SLC15A4 sense and transport muramyl dipeptides (MDP), a structural component of gram-positive bacteria, and recruit NOD1 and NOD2 to endolysosomes for downstream signaling events in dendritic cells (DCs) (Nakamura et al., 2014).

Strikingly, prolonged LPS-TLR4 signaling can also promote antigen cross-presentation by downregulating phago-lysosome fusion to hinder phagosome maturation, acidification and degradation (Gil-Torregrosa et al., 2004; Savina et al., 2006, 2009; Alloatti et al., 2015; Lee J.W.et al., 2018). This is accomplished in part by stimulating the Rab34 GTPase, which consequently promotes perinuclear clustering of lysosomes to reduce phagosome-lysosome fusion (Alloatti et al., 2015). The toggle between MHC-II vs. MHC-I may also depend on TFEB in dendritic cell. CD11c^+^ CD8α^+^ dendritic cells, which prefer MHC-I antigen presentation to CD8+ T cells, express much lower TFEB expression compared to CD11c^+^ CD4^+^ dendritic cells, then prefer MHC-II-mediated presentation (Samie and Cresswell, 2015). Consistent with this, over-expression of TFEB in dendritic cells promoted MHC-II-mediated antigen presentation, but impaired cross-presentation by MHC-I (Samie and Cresswell, 2015). Finally, TFEB forms a positive feedback loop to express MCOLN1 to coordinate dendritic cell migration, macropinocytosis and antigen presentation (Bretou et al., 2017). Specifically, bacterial sensing causes TRMPL1-mediated lysosomal Ca^2+^ efflux to stimulate myosin-II at the rear of the cell, accelerating cell migration. This axis is potentiated by repression of macropinocytosis, which likely reduces mTORC1 activity, boosting TFEB and leading to increased TRPML1 expression (Vargas et al., 2016; Bretou et al., 2017). As a corollary, dendritic cells disrupted for TRPML1 or TFEB display less persistent and slower migration trajectories due to mis-regulation of F-actin across chemokine gradient upon LPS stimulation (Bretou et al., 2017). These surprising observations evince the power of the lysosome to sense, integrate and modulate various cellular systems to coordinate complex responses like migration to infection sites and coordinate antigen presentation.

### Lysosome-Mediated Membrane Repair

The plasma membrane repair response relies on lysosomal exocytosis and subsequent endocytosis of damaged plasma membrane in order to reseal and repair it (Reddy et al., 2001; Idone et al., 2008). In part, this happens because lysosomal exocytosis releases lysosomal proteases that then help remodel the extracellular matrix, which aids in membrane repair (Castro-Gomes et al., 2016). Indeed, RNAi screens identified lysosomal cathepsins B, L, and D as well as ASM as key players in the membrane repair response. It is speculated that lysosomal proteases help gain access to the site of injury by cleaving and clearing cell surface proteins as well as the extracellular matrix (Castro-Gomes et al., 2016). Moreover, activation of ASM also converts the membrane lipid sphingomyelin on the plasma membrane into ceramide. This creates a membrane domain rich in ceramide in proximity to the site of injury. Ceramide rich domains are known to cause inward budding of the membrane and subsequent endocytosis. Consequently, this leads to the endocytosis of the damaged membrane and subsequent resealing of the membrane (Tam et al., 2010; Castro-Gomes et al., 2016). Interestingly, lysosome exocytosis and ASM were also shown to stimulate rapid endocytosis after sonoporation with ultrasound activated microbubbles (Fekri et al., 2016).

As described above, damaged organelles like lysosomes are labeled by galectins, which locally abate mTORC1 activity, stimulate AMPK, and label the offending organelle for autophagic clearance (Kumar et al., 2017; Jia et al., 2018, 2019). More precisely, this can be partitioned into two parts. First, combined loss of mTORC1 and gain of AMPK stimulates ULK1 to promote autophagy initiation around the damaged lysosome. Second, galectins specify damaged organelles by binding to TRIMs, which are autophagic receptors that help the autophagic machinery precisely act on damaged organelles. For example, Gal3 interacts with TRIM16, which then binds ATG16L1, ULK1, and BECN1 to promote autophagophore formation and growth around the target organelle (Kumar et al., 2017). TRIM16 in cooperation with Gal3 can also form complexes with regulators of mTORC1, TFEB and calcineurin to promote nuclear translocation of TFEB, induce autophagic gene expression to further adapt the cell to organelle damage (Chauhan et al., 2016; Kumar et al., 2017). Overall, lysosomes ultimate aid in the resolution of surface and internal membrane damage by exocytosis and autophagy.

## Concluding Remarks and Key Challenges

Lysosomes are classically viewed as terminal degradative organelles along autophagic, phagocytic and endocytic pathways. Yet, lysosomes have emerged as dynamic signaling hubs that sense, integrate and generate responses to stresses and other conditions. In this review, we focused on key nutrient, energy, infection and membrane damage triggers that are interpreted by lysosome-based machinery and the functional outputs they regulate including autophagy, metabolic activity, inflammation, immune adaptation and response, and damage resolution and clearance. Our aim was to broadly illustrate how lysosomes compartmentalize and integrate stress inputs and cellular response. In balancing the choice of topics and molecular details, we acknowledge that this review is not exhaustive and sacrifices many details about the topics that we did opt to discuss; given this, we express our sincere apologies to the authors from whom much has been learned from but that were not cited here. In addition, there remains much to be learned about how lysosomes sense, integrate, and elicit cellular responses. In particular, while individual pathways are complex onto themselves, there is a large degree of cross-talk between these pathways as evinced by mTORC1, AMPK, and GSK3. Predicting cellular decisions “by hand” when all these pathways are taken into account is a major challenge, if not impossible. Thus, the development of mathematical and computational models will be critical to help predict how all these molecular algorithms elicit cellular decisions. Examples of these approaches have been described recently for mTORC1 signaling (Rahman and Haugh, 2017; Sulaimanov et al., 2017; Varusai and Nguyen, 2018). For instance, Varusai and Nguyen employed mathematical modeling to better understand how DEPTOR protein levels, a common inhibitory subunit of mTORC1 and mTORC2 can generate complex feedback loops (Varusai and Nguyen, 2018). Second, while we chiefly treated lysosomes as a uniform entity in this review, we also remarked that lysosomes are a heterogeneous population. Given this, it is not known if specific lysosome sub-populations are preferentially involved in sensing and governing specific stresses and pathways. Finally, while we now appreciate that lysosomes are able to adapt to various cues, it is not clear if distinct cues leads to specific lysosomal adaptation programs; for example, activation of TFEB may lead to preferential expression of gene sub-groups depending on how TFEB is stimulated. Overall, much remains to be understood about how lysosomes serve to sense, organize and elicit responses to a variety of extracellular and intracellular cues, and how these are co-opted by patho-physiological processes like infection and cancer.

## Author Contributions

Both authors listed have made a substantial, direct and intellectual contribution to the work, and approved it for publication.

## Conflict of Interest Statement

The authors declare that the research was conducted in the absence of any commercial or financial relationships that could be construed as a potential conflict of interest. The handling Editor declared a past co-authorship with one of the authors RB.

## Abbreviations

4E-BP1: eukaryotic translation initiation factor 4E-binding protein 1
ALR: autophagic lysosome reformation
AMPK: AMP-activated protein kinase
ASM: acid sphingomyelinase
ATG: autophagy regulated gene
CLEAR network: coordinated lysosomal expression and regulation gene network
DC: dendritic cell
EEA1: early endosome antigen 1
ER: endoplasmic reticulum
ESCRT: endosomal sorting complex required for transport
FBP: fructose-1,6-bisphosphate
FLCN: folliculin
FOXO: forkhead box transcription factors class O
GAL: Galectin
GAP: GTPase activating protein
GEF: guanyl exchange factor
GSK3: glycogen synthase kinase 3
HOPS: homotypic fusion and proteins sorting complex
LAMP1: lysosomal associated membrane protein 1
LAMTOR: lysosomal adaptor and mTOR activator
LDL: low density lipoprotein
LKB1: liver kinase B1
LPS: lipopolysaccharide
MHC: major histocompatibility complex
mTORC1: mechanistic target of rapamycin complex 1
NOD: NOD like receptors
PAT1: proton-assisted amino acid transporter-1
PIP_3_: phosphatidylinositol-3,4,5-trisphosphate
PPARα: peroxisome proliferator activated receptor alpha
RILP: rab interacting lysosomal protein
RSK: ribosomal s6 kinase
S6K1: ribosomal protein s6 kinase-1
SNARE: SNAP receptor
SREBP: sterol regulatory element-binding protein
STUB1: STIP1 homology and U-box containing protein 1
TFEB: transcription factor EB
TLR: toll like receptor
TRPML: transient receptor potential channel mucolipin 1
TSC1/2: tuberous sclerosis complex 1/2
ULK1: Unc-51 like autophagy activating kinase
V-ATPase: vacuolar ATPase H^+^ pump
ZKSCAN3: zinc finger with KRAB and SCAN domains 3
